# The role of affective control, strategy repertoire and subjective emotion regulation success in developmental internalising psychopathology

**DOI:** 10.1038/s41598-024-72336-9

**Published:** 2024-09-11

**Authors:** Carolin C. L. M. Herber, Lea L. Lott-Sandkamp, Elisa R. Straub, Brunna Tuschen-Caffier

**Affiliations:** 1 Department of Clinical Psychology and Psychotherapy, Institute for Psychology, University of Freiburg, Engelbergerstr. 41, 79106 Freiburg, Germany; 2 Department of Psychology, Cognition, Action, and Sustainability, University of Freiburg, Freiburg, Germany

**Keywords:** Human behaviour, Psychology

## Abstract

Adolescence poses significant challenges for emotion regulation (ER) and is thus a critical phase in the emergence of various mental disorders, specifically internalising disorders such as anxiety and depression. Affective control, defined as the application of cognitive control in affective contexts, is crucial for effective ER. However, the relationship between ER and affective control is unclear. This study examined the predictive role of ER strategies and difficulties in affective control, measured as the congruency effect and error rate on an Emotional Stroop task (EST), in a sample of adolescents and young adults (aged 14–21, *M* = 17.28, 22% male). It was hypothesised that participants with internalising disorders would show higher congruency effects and error rates on the EST than healthy controls after a psychosocial stress induction, indicating lower affective control. Surprisingly, our findings revealed no significant differences in these measures between the groups. However, higher depression scores were associated with increased EST errors. While ER strategies and difficulties did not predict affective control, exploratory analyses unveiled associations between depression scores and ER strategy repertoire, perceived ER success and the ER strategy Acceptance. These findings underscore the importance of implicit ER facets, particularly perceived ER success and flexibility to change between applied strategies for adolescents and young adults with elevated depressive symptoms.

## Introduction

Adolescence is characterised by significant cognitive, emotional, hormonal, physical, and social changes^1^. During this phase, adolescents face various challenges, which include assuming more mature social roles that entail increased responsibilities and the need for independence, navigating peer and romantic relationships, and transitioning relationships with parents as they seek greater autonomy. These challenges place substantial demands on emotion regulation^2^, making adolescence a critical phase in the emergence of various mental disorders, specifically internalising disorders such as anxiety and mood disorders^3^. One self-regulatory process that is considered crucial in the development of effective emotion regulation is affective control^4^.

Affective control describes the application of cognitive control in affective contexts, that is, the ability to flexibly orient one’s attention and allocate cognitive resources to goal-relevant affective information while disengaging from irrelevant affective information^4^. For example, affective control enables us to maintain focus on studying in the library despite our friends making jokes in the background. Over the course of development, affective control appears to mature in a non-linear fashion, as research suggests that affective control is reduced during adolescence compared to childhood and adulthood, possibly creating a developmental window with increased vulnerability towards negative affective stimuli (for a comprehensive review, see^4^). This notion is in line with the findings of a large-scale meta-analysis of behavioural and neuroimaging data that identified deficits in affective control as a trans-diagnostic factor in mental disorders^5^. Specifically, impairments in affective control have been associated with internalising mental disorders^6,7^.

At the same time, these disorders have been linked to aberrant emotion regulation [ER;^8–10^]. ER can be broadly defined as an individual’s internal and external processes to modulate their emotional reactions in order to accomplish their goals^11^. For instance, higher levels of anxiety symptoms in adolescents were associated poorer emotional clarity (i.e., the ability to make sense of emotional responses), higher non-acceptance of emotions (i.e., unwillingness to accept certain emotional responses) as well as a more negative self-evaluation of one’s own ability to manage emotions^12^. Furthermore, higher levels of social anxiety and depression symptoms in adolescents were associated with decreased emotional awareness (i.e., the ability to recognize and understand one’s own emotions) and limited access to ER strategies^13^. Limited access to ER strategies and overall difficulties in trait ER also predicted depressive symptoms in adolescents, both cross-sectionally and longitudinally over a timespan of two years^14^. In addition, the choice of ER strategies in adolescents has been linked to psychopathology. A meta-analysis integrating 35 studies showed that adolescents with anxiety and depressive symptoms engaged in less cognitive reappraisal (i.e., the ability to reinterpret a situation to alter its subjective meaning), acceptance (i.e., allowing oneself to experience an emotion without attempting to change it), and problem-solving^15^. The utility and adaptiveness of distraction as an ER strategy is highly context dependent, as it can be applied in terms of positive refocusing or experiential avoidance^16^. Apart from the specific ER strategies applied, other factors tied to ER have been proposed to influence ER success, namely perceived ER success as a facet of ER-specific self-efficacy (i.e., one’s own beliefs about being able to achieve or having achieved ER success;^12,17^) as well as ER flexibility (i.e., the ability to evaluate contextual demands, have a range of strategies to choose from and modify the applied strategy based on its effectiveness as defined in the framework by Bonanno and Burton;^18,19^).

Although both affective control and ER have been linked to psychopathology and specifically internalising mental disorders, their relationship is poorly understood. The cognitive neuroscience perspectives of ER states that cognitive control processes that include affective control are vital for successful regulation of negative emotion^20^. What affective control and ER have in common is that they are both relying on someone’s goals and motivation^21^ and that abilities in one area are predictive of abilities in the other^22^. However, what components of affective control and ER are related to each other and how their interaction impacts internalising psychopathology comprises a gap in the research literature.

This gap in understanding their relationship could, in part, be attributed to previous approaches in assessing affective control. Affective control is commonly studied using the Emotional Stroop task (EST,^23,24^, for a meta-analysis see^25^), an adaptation of the classic Stroop task^26^. During the EST, participants view images of facial expressions depicting different emotions with an emotional word (e.g., “happy” or “fear”) printed across the face. Participants are instructed to identify the emotion expressed through the face whilst ignoring the emotion word. Depending on whether the emotion of the face and the word match, trials are marked as congruent or incongruent. Since incongruent trials contain an image and a word of different emotions causing semantic conflict, participants must exert affective control to inhibit their automatic tendency to read the word. As affective and cognitive information compete for processing resources^27^, the presence of incongruent affective information hampers cognitive processing, which manifests as longer reaction times (RT) and increased errors in incongruent trials relative to congruent trials. This phenomenon is called the congruency effect or Stroop interference effect^28^. The congruency effect in the EST represents an individual’s attentional bias towards emotional stimuli^29^ that manifests in failures in emotional control and has been found to be comparable across clinical and healthy participants^30^. A recent meta-analysis of the EST in depression and anxiety disorder found greater interference by diagnosis-related and negative stimuli, but not by positive stimuli^31^. When it comes to incongruent trials, individuals with high anxiety responded slower than individuals with low trait anxiety during incongruent trials showing a neutral face and a fearful word compared to a fearful face and a neutral word, suggesting a bias to fearful words in anxious subjects^32^.

Although the EST offers a robust way to assess affective control, and its individual task performance has been linked to ER, it remains unclear which aspects of ER difficulties and strategies are specifically linked to the EST. Two of these aspects are response inhibition and initiation to emotional contents, which develop linearly with ER in childhood and adolescence^33^. While cognitive and affective control abilities increase with age with a diminished cognitive performance in late adolescence^34^, the ability to regulate emotions also increases. Adolescents rely more on cognition-related ER strategies such as cognitive reappraisal and acceptance, while children rely more on behaviour-related ER strategies such as distraction (i.e., behavioural preoccupation with something else to not think about the emotion-eliciting stimulus) and are less able to switch between strategies, representing lower ER strategy repertoire and potentially lower ER flexibility^35,36^. To the best of our knowledge, this is the first study to look at ER as a multilevel process and to link ER difficulties, ER strategies, ER success and ER strategy repertoire with affective control in an experimental setting in young adults and adolescents with anxiety and depression.

In the present study, we investigated the relationship between affective control, operationalized as the congruency effect and the number of errors on the EST, and ER in adolescents and young adults (for simplicity, they will both be referred to as youth) with internalising disorders. First, we hypothesised that (i) youth with internalising mental disorders (an anxiety disorder and/or depression) would exhibit lower affective control compared to healthy youth. Second, we explored whether ER strategies and difficulties predict affective control following a stress induction task and hypothesised that across groups (ii) more cognitive reappraisal, distraction and a higher subjective success in implementing those strategies would positively predict affective control. Third, we explored which aspects of ER would predict affective control and hypothesized that (iii) higher levels of trait ER difficulties and the specific ER difficulties impulse control difficulties, higher lack of emotional awareness and poorer access to emotion regulation strategies would inversely predict affective control. Lastly, we explored the developmental trajectory of affective control and ER and hypothesised that (iv) age would moderate the relationship between the two variables.

## Methods

This cross-sectional study was part of a larger study about emotion regulation and sleep (Herber et al., in preparation, see supplementary materials for a detailed overview of the study procedures) approved by the ethics committee of the University of Freiburg. All study procedures were carried out in accordance with the Declaration of Helsinki. The study was preregistered on As predicted (study number 106298), modifications of the preregistered design are reported in the supplementary materials.

### Sample size calculation

Appropriate sample size for a MANOVA (Global effects) with two groups and three outcome variables was determined based on an a priori power analysis using G*Power (Version 3.1.9.4;^37^). We used an estimated effect of f^2^ = 0.16 based on the large effect size found in a meta-analysis on the Stroop effect in youth with depression^38^, an alpha criterion of 0.05 and a power of 0.80. The results indicated a minimum sample size of *N* = 74.

### Participants

We recruited participants through the city’s population register, youth groups and the outpatient treatment centre of the Child and Adolescent Psychiatric Clinic. Inclusion criteria were German language proficiency (native language or fluent), aged 14–21 and having a current depressive or anxiety disorder (for the CLIN group) or no current or past disorder (for the HC group). Exclusion criteria included the presence of any mental disorder other than depressive or anxiety disorder, a prior psychotic episode, a history of drug or alcohol abuse, acute suicidal ideation, and psychoactive medication intake. The study included a total of 91 participants (22% male). The clinical group (CLIN) with internalising disorder comprised 31 participants (34.07%), half of which met the criteria for a depressive disorder (*N* = 16) and in part a comorbid anxiety disorder, while the other half met the criteria for only an anxiety disorder (*N* = 15). The decision to include a comorbid group was made given the high comorbidity between the two disorder groups (^39^; see supplementary materials for flow chart of study inclusion, group allocation, and proportion of disorder types and comorbidities). The healthy control (HC) group comprised 60 participants who did not have any current or lifetime mental disorder. Group allocation was based on DSM-5 research diagnoses established using the Diagnostic Interview for Mental Disorders (DIPS), either in the short version for older adolescents or in the child version for those in middle adolescence. The Kinder-DIPS is a standardized, open-access interview of mental disorders for German speaking youth^40^ of high interrater reliability^41^ which can be carried out either with the youth alone or including their parents. In this study, the youth were interviewed alone. To analyse age effects, we split the sample into two groups. Approximately half of the participants (*N* = 41, age *M* = 16.00, *SD* = 1.46) were in adolescence, aged between 14 and 18 and went to secondary school, while the other half (*N* = 50, age *M* = 20.08, *SD* = 0.8) was in early adulthood, aged between 19 and 21 and attended University.

### Measures

#### Emotional Stroop task

**Fig. 1:**
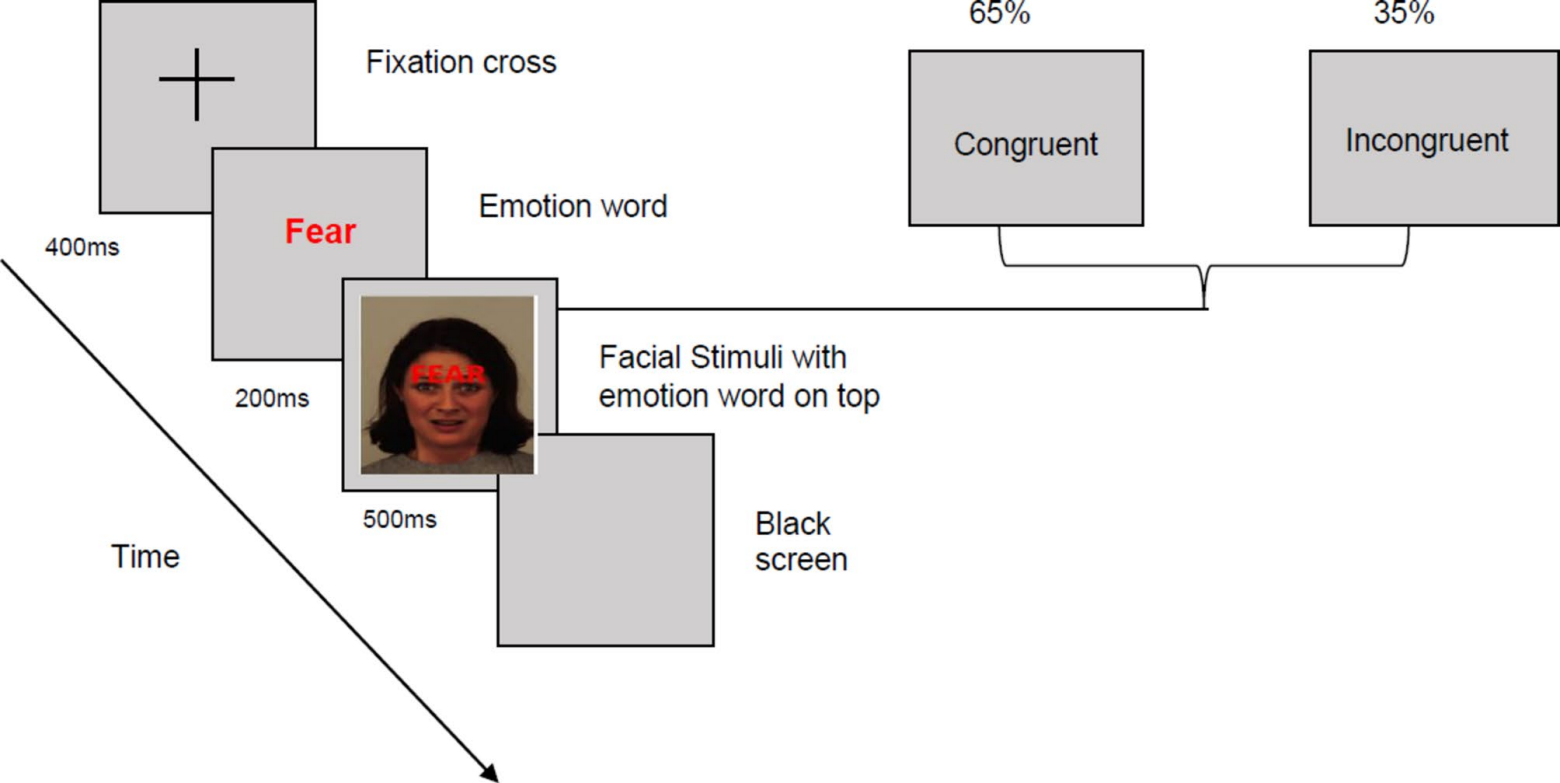
Length and order of stimuli presentation during the EST.

We programmed the EST in E-Prime (Version 2.0). Stimuli were happy and fearful faces from the Karolinska Directed Emotional Faces database^42^. The task contained 96 trials separated into two equal blocks with 65% congruent and 35% incongruent trials each. Trial order was randomised. Each trial began with the presentation of a fixation cross for 400 ms followed by the picture-word stimulus for 2000 ms. Missing responses and responses after 2000 ms counted as errors. Affective control was operationalised as the congruency effect and the error rate. The congruency effect was calculated by subtracting the mean reaction time (RT) on all incongruent trials with correct responses from the mean RT of all congruent trials with correct responses, with higher scores indicating poorer affective control. Error rate was calculated as the proportion of trials with incorrect responses relative to the total trial number (Fig. 1).

#### Stress induction

Psychosocial stress was induced using the Trier Social Stress Test (TSST) in a digital version modified for youth^43–45^. The TSST included two tasks followed by an ER phase (see *Procedures*). First, participants were given the beginning of a story and were asked to continue telling the story for five minutes (speech task). Then, participants were asked to perform mental arithmetic while being evaluated by a jury for another five minutes (math task). The difficulty of the math task was adjusted to the participant’s age. The TSST was carried out digitally via the video conferencing platform Zoom (Version 5.15.3) with the participant being in one laboratory room and the experimenter in another. Participants were informed that two additional jury members (one male, one female) would join the video conference. In fact, two pre-recorded videos of the jury members were inserted as virtual backgrounds in two separate Zoom accounts that were set to mute, as it is usually done for people in a video conference not actively speaking. After the TSST, participants underwent a deception check to ensure they perceived the digital TSST as natural and did not suspect deception regarding the videos of the two not actually present jury members. Participants were asked to indicate on a 5-point Likert scale (1 = “Not at all” to 5 = “Very much”) how interested and concentrated they thought the two jury members were and if they had had doubts about them participating in the video conference in real-time. None of the participants included in this analysis reported having doubted the presence of the two jurors. In addition, a manipulation check was performed to ensure successful psychosocial stress induction (see supplementary materials).

#### ER strategies, success and strategy repertoire

After the ER phase following the TSST, participants were asked which ER strategies they had used during the ER phase and could either give a free response or select a predefined strategy from a list. The predefined ER strategies were cognitive reappraisal, acceptance, distraction, social support, problem solving, mindfulness and “Other” (view supplementary materials for description of predefined strategies). Participants could chose as many strategies as they had used from a list with these descriptions. Free responses were later assigned to one of the predefined strategies or “Other”, if they did not match any of the other strategies for data analysis. In addition, participants were asked to indicate their perceived ER success, that is, how successful they believed they were at regulating their stress by rating their current stress level relative to the stress level at the end of the stress test on a 10-point Likert scale (1 = no difference to stress level since end of TSST, 10 = achieved complete relaxation). ER strategy repertoire was dummy coded to measure whether participants used one (0) or multiple (1) strategies.

#### Psychometrics

Before the TSST, participants filled in a series of questionnaires. Habitual ER ability was quantified using the Difficulties in Emotion Regulation Scale (DERS), a 36-item self-report questionnaire assessing the subdimensions *Lack of emotional awareness*, *Lack of emotional clarity*, *Non-acceptance of emotions*, *Limited access to regulation strategies*, *Impulse control difficulties* and *Difficulties engaging in goal-directed behaviour* when distressed (^46^; German Version^47^]). Depressive symptom severity was quantified using the Beck Depression Inventory II (BDI-II;^48^), a questionnaire with 21 items assessing the occurrence of different depressive symptoms within the last two weeks, with higher scores indicating greater levels of depression. The BDI-II can be used for youth aged 13 and above and was found to be of high validity^49^. Anxiety was quantified using the State Trait Anxiety Inventory (STAI), a questionnaire with 20 items assessing state anxiety and 20 items assessing trait anxiety, with higher scores indicating greater anxiety that has also been validated in an adolescent sample^50^. However, there has been criticism pointing out that the STAI trait scale is particularly elevated in depressed individuals and should therefore be seen as a nonspecific measure of negative affectivity^51^.

### Procedures

The present study comprised two sessions. Prior to participation in the lab study, participants were screened for eligibility over the phone. In the first session, participants and their guardians gave their informed consent and participants took part in the diagnostic interview. In the second session, participants filled in the questionnaires before undergoing the TSST. During the subsequent ER phase, participants were instructed to do whatever helped them reduce their stress and relax for the next ten minutes in the laboratory. Afterwards, data on ER strategies and ER success was collected. Finally, participants completed the EST before being debriefed.

### Statistical analyses

Prior to data analysis, statistical assumptions were validated. For the EST, we calculated mean RTs across correct trials, the congruency effect (i.e., mean RT differences between incongruent and congruent trials) and error rates (i.e., the proportion of incorrect trials relative to all 96 trials). To check for the presence of the congruency effect and potential effects of facial stimulus type (= manipulation check for the EST), two 2 × 2 mixed model ANOVAs were calculated using the within-subject factors *congruency* (congruent vs. incongruent) and *facial stimulus* (happy vs. fearful) and the between-subject factor group (CLIN vs. HC). The first ANOVA used the RTs as a dependent variable; the second ANOVA used the error rate. To test the first hypothesis, a MANOVA was computed to compare affective control (quantified as the magnitude of the mean RT, congruency effect and error rates in the EST) between the CLIN and HC group. The factor age (adolescents/young adults) was added as a fixed factor to explore potential age effects. A chi-square goodness of fit test showed that participant sex was unevenly distributed between the two groups (χ^2^[1[= 4.15, *p* = .042, φ = 0.42), thus sex was added as a covariate. Since detecting a group difference would have required a bigger sample size to be adequately powered (which was not possible due to the complexity of recruitment and financial restraints of the study), we decided to include regression analyses of dimensional measures of internalising symptoms as secondary analyses. Then, we computed two multiple linear regression analyses using the entry method with the two measures of affective control (congruency effect, error rates in the EST) as criteria to test our second and third hypothesis. Added predictors were the ER strategies *Distraction* and *Cognitive reappraisal*, ER success and ER strategy repertoire and for the second hypothesis. These strategies were chosen over the other strategies based on previous studies that tested them in the EST^52^. For the third hypothesis, the predictors were the ER difficulties *Trait ER difficulties*, *Impulse control difficulties*, *Limited access to ER strategies* and *Lack of emotional clarity,* as their conceptual framework seemed to overlap with the one of affective control.

## Results

### Affective control between internalising disorders and healthy participants

**Fig. 2:**
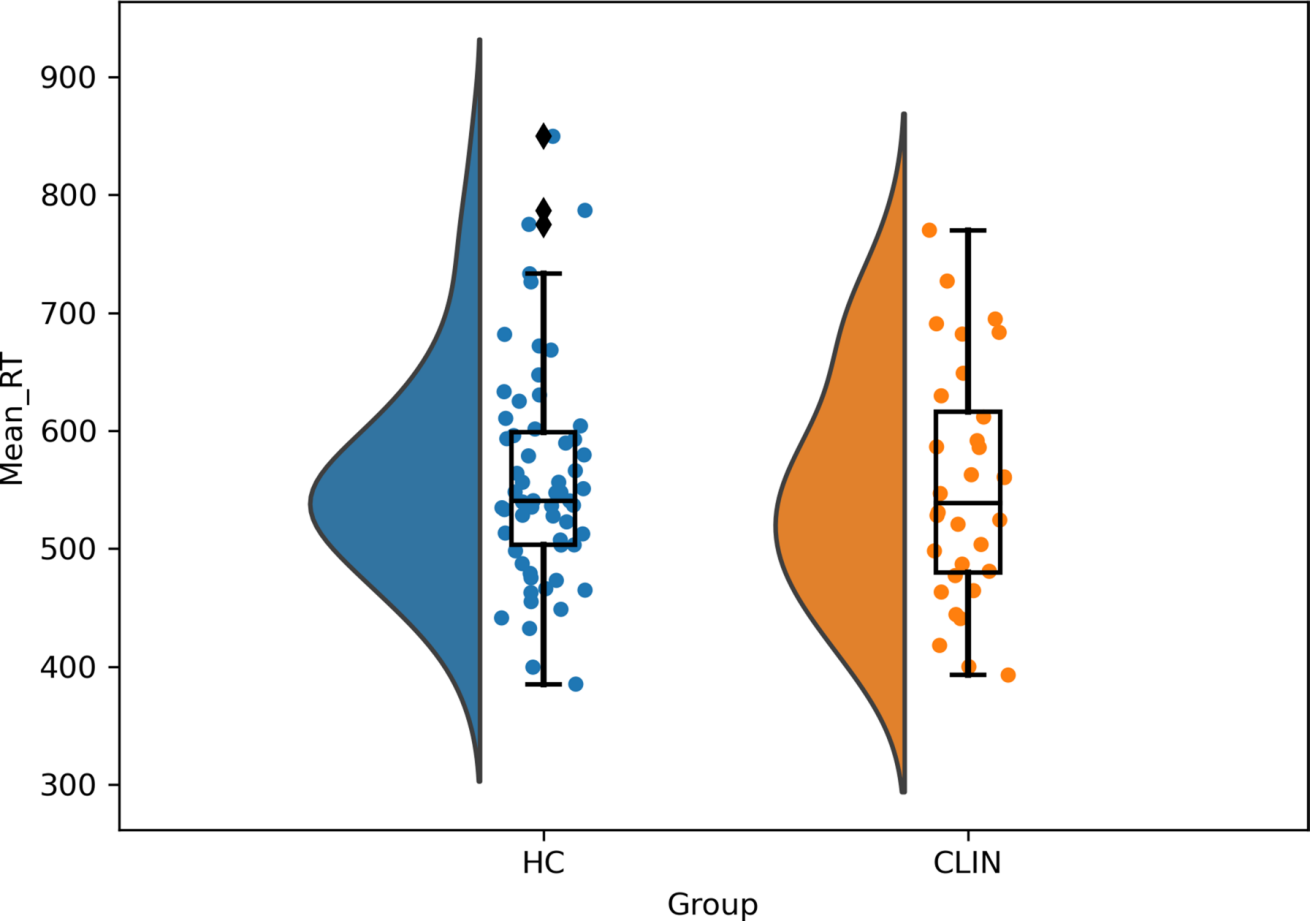
Half violin plots of the congruence effect in the EST of the CLIN and HC groups.

A one-way MANOVA was carried out to test if participants with internalising mental disorders would exhibit lower affective control compared to healthy youth. We detected no statistically significant differences between the CLIN and the HC group on the mean RT (see Fig. 2), congruency effect and error rates, *F*(3, 85) = 0.634, *p* = .60, Wilk’s Λ = 0.978. However, a significant difference was detected between adolescents and young adults on the performance on the EST, *F*(3, 85) = 7.14, *p* < .001, Wilk’s Λ = 0.799. There was no significant interaction effect between the clinical groups and the age groups *F*(3, 85) = 0.06, *p* = .98, Wilk’s Λ = 0.998, and no significant effect of sex, *F*(3, 85) = 0.063, *p* = .94, Wilk’s Λ = 0.998.

### Dimensional approaches to psychopathology and their effect on affective control

#### Trait and state anxiety on affective control

When looking at anxiety as a dimensional variable as scores of state and trait anxiety on the STAI, neither trait anxiety (*B* = − 0.21, *SE* = 0.41, *ß* = − 0.06, *p* = .614) nor state anxiety (*B* = − 0.17, *SE* = 0.51, *ß* = − 0.04, *p* = .747) could predict the congruency effect *R*^*2*^ = 871.12, *F*(2,89) = 0.22, *p* = .800. Similarly, neither trait anxiety (*B* = 0.23, *SE* = 0.2, ß = 0.05, *p* = .251) nor state anxiety (*B* = − 0.2, *SE* = 0.25, *ß* = − 0.09, *p* = .414) could predict the error rate on the EST *R*^2^ = 897.15, *F*(3,88) = 0.65, *p* = .587.

#### Depressive symptoms on affective control

The depressive symptoms along the BDI-II (*B* = − 0.1, *SE* = 0.47, *ß* = − 0.02, *p* = .829) did not predict the congruency effect *R*^*2*^ = 90.08, *F*(1,91) = 0.05, *p* = .829. The BDI-II score (*B* = 0.21, *SE* = 0.09, *ß* = 0.23, *p* = .029) did however predict the error rate *R*^*2*^ = 0.21, *F*(1,90) = 4.71, *p* = .029 as demonstrated in this secondary analysis.

### Effects of ER strategies and difficulties on affective control

To analyse the effects of ER strategies (hypothesis 2) and ER difficulties (hypothesis 3) on the congruency effect and error rate in the EST as two measures of affective control, we computed two multiple linear regression models using the enter method. Both models testing the effect of the ER strategies failed to reach significance (congruency effect: *F*[3, 87] = 1.18, *p* = .32, *R*^2^ = 0.039, adjusted *R*^2^ = 0.01; error rate: *F*[3, 87] = 0.02,* p* = 1.00, *R*^2^ = 0.001, adjusted *R*^2^ =  − 0.03). Similarly, both models testing the effect of ER difficulties failed to reach significance (congruency effect: *F*[3, 87] = 1.90, *p* = .16, *R*^2^ = 0.06, adjusted *R*^2^ = 0.03; error rate: *F*[4, 86] = 0.16, *p* = .96, *R*^2^ = 0.07, adjusted *R*^2^ = − 0.04).

### Developmental trajectory of affective control

To explore if age would moderate the relation between ER and affective control, we had planned to compute a moderator analysis. We tested the assumptions of moderator analysis (i.e., if age would moderate the relationship between affective control and ER.) by computing the correlation between trait ER and the two affective control measures. As there were no significant correlations between trait ER and the congruency effect (*r*[91] = 0.13, *p* = .2) or the error rate (*r*[91] = 0.14, *p* = .18), we instead compared adolescents and young adults on the two affective control measures within the MANOVA presented before (see section "Affective control between internalising disorders and healthy participants"). To check for a strong disordinal interaction, the moderation analysis itself was still carried out but revealed non-significant results (see supplementary materials).

### Exploratory analyses on links between ER measures and depressive symptoms

**Fig. 3:**
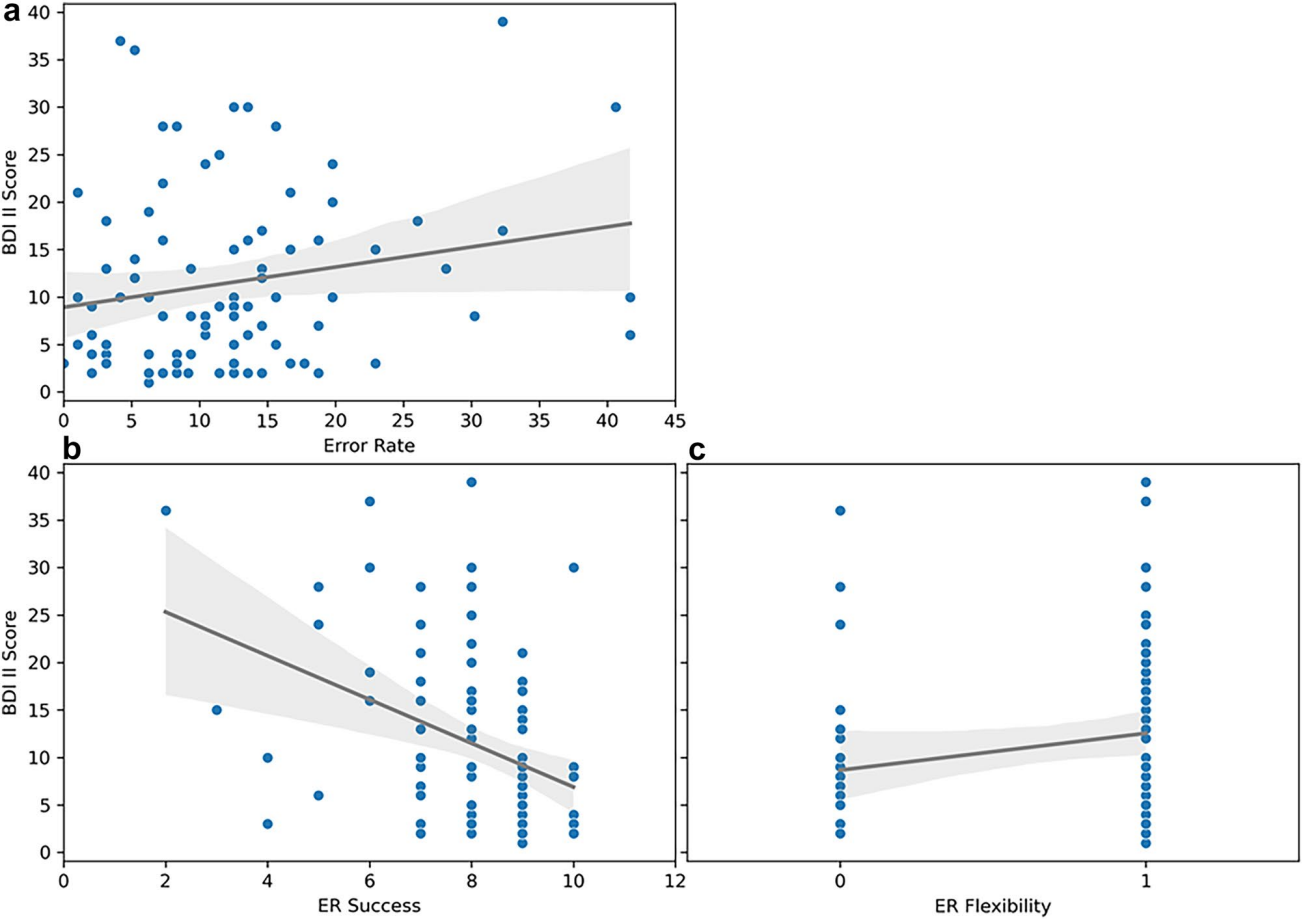
(**a**) Relationship between scores on the BDI-II and the error rate in percentage as a scatterplot. (**b**) Relationship between BDI-II scores and the ER success along a numerical analogue scale. (**c**) Relationship between the BDI-II score and the ER flexibility, with 0 = only one strategy was used and 1 = more than one strategy were used.

As we had found the CLIN group to show not surprisingly significantly higher levels of depression symptoms than the HC group (see Table 1), we exploratorily investigated which factors related to ER and affective control would predict depressive symptoms in our sample. We therefore computed a multiple linear regression analysis using the entry method. The overall model was significant, *F*(7,83) = 5.70, *p* < .001, *R*^*2*^ = 0.325, adjusted *R*^*2*^ = 0.26. The regression showed that the EST error rate, the ER strategy Acceptance, ER strategy repertoire and ER success were all predictive of depressive symptoms (see Table 2), while the congruency effect and the ER strategies Distraction and Cognitive reappraisal were not significant predictors. Higher error rates and higher ER strategy repertoire, respectively, were associated with higher levels of depressive symptoms (see Fig. 3a,c). On the contrary, more frequent use of the ER strategy Acceptance and higher perceived ER success, respectively, were associated with lower levels of depressive symptoms (see Fig. 3b).

**Table 1:** Descriptive statistics of participant age and psychometric data (mean ± standard deviation).

	CLIN group (*n* = 31)	HC group (*n* = 60)	*p*	Cohen's *d*
Age	17.94 ± 2.17	18.40 ± 2.42	.186	0.20
BDI-II score	18.87 ± 9.96	7.73 ± 6.26	< .001	− 1.40
STAI Trait Anxiety	37.10 ± 11.16	39.22 ± 11.8	.205	0.18
STAI State Anxiety	41.26 ± 8.87	40.14 ± 9.37	.292	− 0.12
DERS score	83.42 ± 22.06	85.82 ± 22.31	.335	0.09

CLIN group = clinical group with internalising disorders, HC group = healthy control group. Group differences were explored using two-tailed independent samples *t*-tests.

**Table 2:** Linear regression analysis of affective control and ER measures on depressive symptoms.

Predictor	*b*	*SE*	β	*p*
EST measures				
Congruency effect	− 0.02	0.02	− 0.09	.329
** Error rate**	**0.24**	**0.10**	**0.23**	**.016**
ER strategies				
Distraction	2.09	1.85	0.11	.261
** Acceptance**	**− 3.77**	**1.91**	**− 0.19**	**.050**
Cognitive reappraisal	− 1.43	1.95	− 0.08	.467
**ER strategy repertoire**	**6.78**	**2.27**	**0.32**	**.004**
**ER success**	− **2.34**	**0.55**	− **0.39**	**< .001**

Findings significant at *p* ≤ .05 are highlighted in bold font.

## Discussion

The present study examined the relationship between different components of ER and affective control, operationalized as the congruency effect and the number of errors on the EST, and investigated, if this relationship differed in youth with internalising disorders and healthy counterparts.

First, we had hypothesized that participants with internalising mental disorders would exhibit poorer affective control, manifesting as longer RTs and a larger congruency effect, compared to healthy participants. Contrary to our expectation, we found that the two groups did not differ on the two affective control measures, which diverges from earlier findings suggesting affective control deficits in individuals with internalising mental disorders^31,38^. Although we did not find the expected group differences, which might be explained by inadequate power given the sample size, the EST did capture facets of psychopathology in our sample. Our exploratory findings showed that error rates in the EST positively predicted the level of depressive symptoms across groups, but not symptoms of anxiety. On one hand, the findings regarding depressive symptoms are in line with previous studies findings more errors and slower RTs in depressed subjects^53^. On the other hand, our findings regarding anxiety symptoms contradict those of previous studies suggesting that higher levels of trait anxiety were related to slower RTs and more errors on a facial Stroop task^31^. In light of these discrepancies, several potential factors are to be discussed. It is possible that psychopathology-related effects are obscured by methodological differences between our study and previous studies, such as the choice of visual stimuli. Meta-analytic findings revealed greater congruency effects in adults with depression compared to healthy controls when presented with mood-congruent negative stimuli^38^. Although the meta-analysis did not differentiate further within the negative-valence category (i.e., angry versus sad faces), it has been hypothesized that only specific stimuli elicit attentional biases specific to psychopathology, in that depressed individuals exhibit an attentional bias when they are viewing sad faces^54^. However, the series of experiments conducted by^54^ revealed no significant attentional biases regardless of the valence of the stimulus in adults with depression despite adequate statistical power, which is consistent with our findings. It is thus unlikely that the choice of emotional stimulus alone camouflaged potential psychopathology-related effects. Instead, the age of our clinical group might play a role, as the vast majority of prior studies that explored psychopathology-related effects on affective control recruited adult samples (see meta-analysis by^31^). Since our study is one of the first to experimentally explore affective control and ER in youth with only a few studies having looked at this association before^55–58^, with a previous review suggesting that ER success in adolescence relies on the maturation of affective control^4^, it is unclear to which extent other findings from adult samples can be generalized to younger individuals still in development. One explanation for a difference between adolescents and adults might be that this attentional bias to mood-congruent words for depressed adult subjects correlates with fMRI data of increased activity in an area of the prefrontal cortex, the rostral anterior cingulate cortex^28^, which has been found to develop at a different pace in depressed compared to healthy adolescents^59^. Due to the lack of longitudinal data, it remains unknown whether the effects of psychopathology on affective processing are immediate or whether they manifest gradually over time, which would explain why we did not observe them in our young sample.

Second, we had investigated whether ER strategies and subjective ER success would predict affective control following a psychosocial stress induction. We had hypothesized that more cognitive reappraisal, distraction and a higher subjective ER success in implementing those strategies would positively predict affective control across groups. Contrary to our hypothesis, we found that the choice of ER strategy and ER success did not predict affective control. This contradicts Pruessner and colleagues^19^ notion that ER is closely linked to cognitive control, although one should note that their perspective focuses on the relevance of cognitive control for ER flexibility in particular. However, there is initial evidence that cognitive control should not only influence ER flexibility but also ER strategies. For instance, individuals with a higher working memory capacity were found to use cognitive reappraisal more often than those with lower working memory capacity^60^. However, the relationship between cognitive control and different ER facets such as strategy repertoire or subjective ER success remains unknown, highlighting the need for further research in this area.

Third, we had analysed what difficulties in ER would predict affective control and had hypothesized that higher levels of trait ER difficulties, impulse control difficulties, higher lack of emotional awareness and poorer access to ER strategies would inversely predict affective control. In our study, ER difficulties did not predict affective control, which diverges from our hypothesis and the established literature. For instance, O’Bryan and colleagues^61^ found links between affective control and better emotional clarity and a higher ability to engage in goal-directed behaviour during negative affect. At the same time, they failed to find links with impulse control difficulties, emotional acceptance and access to ER strategies, suggesting that only specific aspects of ER difficulties might show associations with affective control. The authors noted that better attentional control might allow for more emotional clarity by reducing threat-related attentional biases and increasing one’s engagement in goal-directed behaviour when upset, thus preventing getting absorbed by negative emotions. As the relationship between ER difficulties and affective control remains poorly understood, further research should investigate potential links in more detail.

Forth, we had assessed the developmental trajectory of affective control and ER and had hypothesised that age would moderate the relationship between the two variables. In our study, participant age did not appear to moderate the relationship between affective control and ER. This might suggest that the two constructs do not develop linearly throughout adolescence but at different rates. However, age did have an effect on performance on the EST, with more errors in adolescents than in young adults. This finding is in line with previous studies describing higher error rates in adolescents compared to young adults on affective control tasks^34,62^, suggesting that neural circuits involved in the processing of emotional stimuli undergo significant changes throughout adolescence (for an overview, see^63^). This could underline the vulnerability of adolescence as a developmental stage given it is often the onset time of mental disorders that carry on into adulthood with a peak age of onset at 14.5 years^64^.

Exploratory analyses revealed that the error rate, the ER strategy Acceptance, ER success and ER strategy repertoire significantly predicted depression symptoms. Higher error rates and higher ER strategy repertoire, respectively, were associated with higher levels of depressive symptoms. Higher error rates in a spectrum of cognitive tasks have previously been linked to higher levels of depression in adults^65,66^, possibly reflecting psychopathology-related alterations in brain function that have their onset during adolescence (for a review, see^67^). Regarding ER strategy repertoire, our finding is surprising, as higher strategy repertoire and therefore higher ER flexibility has previously been associated with lower but not higher levels of depressive symptoms^68,69^. Those studies differed from our investigation in that they used a self-report questionnaire to determine trait ER flexibility. In contrast, our study assessed only the strategy repertoire component of ER flexibility by asking participants to report which strategies they had actually applied after the psychosocial stress induction. As a result, both measures capture different facets of ER flexibility. Another interpretation might be that subjects that had higher depressive scores were not as effective in either implementing the strategy that they had initially picked or that that strategy was not sufficient for their self-regulation hence they switched, while those without depressive symptoms were successful with their single strategy and did not require switching. While trait ER refers more to the breadth of an individual’s ER strategy repertoire, state ER refers to the ability to evaluate contextual demands and the ability to choose and modify ER strategies accordingly^18^. As a result, ER flexibility can promote stress resilience and indirectly protect against mental health problems^70^, but only when strategy switching is actually effective for ER. Increased strategy switching in adolescent girls has been associated with them experiencing the interpersonal stressor to be more uncontrollable, the intensity of the elicited negative emotion and having the possibility to co-regulate with a peer^71^. The same authors discussed whether this increased likelihood to switch strategies in older adolescents compared to younger adolescents might reflect age-related increases in underlying processes like affective control.

Regarding ER strategy use and ER success, we found that more frequent use of the ER strategy Acceptance and higher perceived ER success, respectively, were associated with lower levels of depressive symptoms. Previous studies in depressed youth have shown that they relied less on adaptive ER strategies such as Acceptance and more on maladaptive strategies such as Rumination^72^. Although this might suggest that specific ER strategies act as protective or risk factors and are thus prognostic for depression in youth, longitudinal studies are needed to determine the causal relationship between ER strategies and psychopathology, as depression onset could trigger a shift in affective processing and thus influence facets of ER such as strategy use and repertoire. Regarding ER success, we found individuals with higher levels of depressive symptoms to be less likely to perceive their ER as successful, suggesting lower ER self-efficacy. This observation corresponds with findings from a longitudinal study in young adults that linked lower ER self-efficacy to higher levels of depression six months later^72^. These findings specific to ER mirror longitudinal data that linked lower self-efficacy, conceptualized on a broader level, to higher levels of depression in youth, young adults as well as across the lifespan^73^. Although this might hint at a particular significance of ER self-efficacy for depression risk, more research on this matter is needed. To sum up, the role of different ER strategies, ER strategy repertoire and ER success in psychopathology should be further investigated, since deeper insights into this matter might help to identify potential targets of psychotherapeutic interventions.

While our study provides novel insights into affective control and ER in youth with internalising disorders, our findings are subject to methodological limitations that should be addressed in future research. First, we did not systematically manipulate which ER strategies participants applied but rather assessed spontaneous strategy use. While this approach offers higher ecological validity and simultaneously allows for measuring the strategy repertoire component as a facet of ER flexibility, it does not control for interindividual variability in other factors that could influence spontaneous strategy use. For instance, research suggests that subjective stressor intensity plays a part in the selection of specific ER strategies^74,75^. Although all participants in our study underwent a standardizes psychosocial stress induction, it is possible that they perceived the paradigm differently and hence chose different ER strategies. Thus, future research is needed to systematically explore which factors determine ER strategy choice and which differential effects specific ER strategies have. Second, we measured ER difficulties and ER success using self-reports. Until this day, it is unclear to what extent self-reports reflect actual biological processes associated with ER, especially in youth, as previous studies yielded mixed results on this matter^76,77^. Fruitful insights could thus come from incorporating psychophysiological or neural measures of ER to better understand the psychobiological links between ER and psychopathology.

Taken together, our findings emphasize the importance of different implicit facets of ER for mental health during adolescence and young adulthood. Gaining a better understanding of the cognitive and neural processes involved in affective processing is crucial for developing targeted prevention programmes and psychotherapeutical treatments for youth at high risk for or affected by depression and anxiety.

## Supplementary Information

Supplementary Information.

## Data Availability

The datasets used and/or analysed in the current study will be made available without reservations from the corresponding author on reasonable request.

## References

[1] Rice, F. P., & Dolgin, K. G. (2005). The adolescent: Development, relationships and culture. *Pearson Education New Zealand*. Boston: Allyn & Bacon.

[2] Nigg, J. T. Annual Research Review: On the relations among self-regulation, self-control, executive functioning, effortful control, cognitive control, impulsivity, risk-taking, and inhibition for developmental psychopathology. *J. Child Psychol. Psychiatry* **58**(4), 361–383. 10.1111/jcpp.12675 (2017). 28035675 10.1111/jcpp.12675 PMC5367959

[3] Klein, R. J., Nguyen, N. D., Gyorda, J. A. & Jacobson, N. C. Adolescent emotion regulation and future psychopathology: A prospective transdiagnostic analysis. *J. Res. Adolesc.* **32**(4), 1592–1611. 10.1111/jora.12743 (2022). 35301763 10.1111/jora.12743 PMC10152987

[4] Schweizer, S., Gotlib, I. H. & Blakemore, S. J. The role of affective control in emotion regulation during adolescence. *Emotion* **20**(1), 80. 10.1037/emo0000695 (2020). 31961183 10.1037/emo0000695 PMC6975522

[5] Schweizer, S. *et al.* The impact of affective information on working memory: A pair of meta-analytic reviews of behavioral and neuroimaging evidence. *Psychol. Bull.* **145**(6), 566. 10.1037/bul0000193 (2019). 31021136 10.1037/bul0000193 PMC6526745

[6] Joormann, J. & Siemer, M. Affective processing and emotion regulation in dysphoria and depression: Cognitive biases and deficits in cognitive control. *Soc. Personal. Psychol. Compass* **5**(1), 13–28. 10.1111/j.1751-9004.2010.00335.x (2011). 10.1111/j.1751-9004.2010.00335.x

[7] De Lissnyder, E. *et al.* Internal cognitive control in clinical depression: General but no emotion-specific impairments. *Psychiatry Res.* **199**(2), 124–130. 10.1016/j.psychres.2012.04.019 (2012). 22578821 10.1016/j.psychres.2012.04.019

[8] Miller, A. B. *et al.* Neural correlates of emotion regulation and adolescent suicidal ideation. *Biol. Psychiatry Cognit. Neurosci. Neuroimaging* **3**(2), 125–132. 10.1016/j.bpsc.2017.08.008 (2018). 29529407 10.1016/j.bpsc.2017.08.008 PMC5851479

[9] McLaughlin, K. A., Hatzenbuehler, M. L., Mennin, D. S. & Nolen-Hoeksema, S. Emotion dysregulation and adolescent psychopathology: A prospective study. *Behav. Res. Ther.* **49**(9), 544–554. 10.1016/j.brat.2011.06.003 (2011). 21718967 10.1016/j.brat.2011.06.003 PMC3153591

[10] Gilbert, K. E. The neglected role of positive emotion in adolescent psychopathology. *Clin. Psychol. Rev.* **32**(6), 467–481. 10.1016/j.cpr.2012.05.005 (2012). 22710138 10.1016/j.cpr.2012.05.005

[11] Thompson, R. A. (1994). Emotion regulation: A theme in search of definition. *Monographs of the society for research in child development*, 25–52. 10.2307/1166137 7984164

[12] Mathews, B. L., Kerns, K. A. & Ciesla, J. A. Specificity of emotion regulation difficulties related to anxiety in early adolescence. *J. Adolescence* **37**(7), 1089–1097. 10.1016/j.adolescence.2014.08.002 (2014). 10.1016/j.adolescence.2014.08.002 25150890

[13] Klemanski, D. H., Curtiss, J., McLaughlin, K. A. & Nolen-Hoeksema, S. Emotion regulation and the transdiagnostic role of repetitive negative thinking in adolescents with social anxiety and depression. *Cognit. Ther. Res.* **41**, 206–219. 10.1007/s10608-016-9817-6 (2017). 28579659 10.1007/s10608-016-9817-6 PMC5455341

[14] Gonçalves, S. F. *et al.* Difficulties in emotion regulation predict depressive symptom trajectory from early to middle adolescence. *Child Psychiatry Human Dev.* **50**, 618–630. 10.1007/s10578-019-00867-8 (2019). 10.1007/s10578-019-00867-8 PMC6589375 30689145

[15] Schäfer, J. Ö., Naumann, E., Holmes, E. A., Tuschen-Caffier, B. & Samson, A. C. Emotion regulation strategies in depressive and anxiety symptoms in youth: A meta-analytic review. *J. Youth Adolescence* **46**, 261–276. 10.1007/s10964-016-0585-0 (2017). 10.1007/s10964-016-0585-0 27734198

[16] Kökönyei, G., Kovács, L. N., Szabó, J. & Urbán, R. Emotion regulation predicts depressive symptoms in adolescents: A prospective study. *J. Youth Adolescence* **53**(1), 142–158. 10.1007/s10964-023-01894-4 (2024). 10.1007/s10964-023-01894-4 PMC10761508 37985558

[17] Gutentag, T., Halperin, E., Porat, R., Bigman, Y. E. & Tamir, M. Successful emotion regulation requires both conviction and skill: Beliefs about the controllability of emotions, reappraisal, and regulation success. *Cognit. Emot.* **31**(6), 1225–1233. 10.1080/02699931.2016.1213704 (2017). 27494261 10.1080/02699931.2016.1213704

[18] Bonanno, G. A. & Burton, C. L. Regulatory flexibility: An individual differences perspective on coping and emotion regulation. *Perspect. Psychol. Sci.* **8**(6), 591–612. 10.1177/1745691613504116 (2013). 26173226 10.1177/1745691613504116

[19] Pruessner, L., Barnow, S., Holt, D. V., Joormann, J. & Schulze, K. A cognitive control framework for understanding emotion regulation flexibility. *Emotion* **20**(1), 21. 10.1037/emo0000658 (2020). 31961173 10.1037/emo0000658

[20] Mueller, S. The influence of emotion on cognitive control: Relevance for development and adolescent psychopathology. *Front. Psychol.* **2**, 12765. 10.3389/fpsyg.2011.00327 (2011). 10.3389/fpsyg.2011.00327 PMC3223617 22275904

[21] Inzlicht, M., Bartholow, B. D. & Hirsh, J. B. Emotional foundations of cognitive control. *Trends Cognit. Sci.* **19**(3), 126–132. 10.1016/j.tics.2015.01.004 (2015). 25659515 10.1016/j.tics.2015.01.004 PMC4348332

[22] Hendricks, M. A. & Buchanan, T. W. Individual differences in cognitive control processes and their relationship to emotion regulation. *Cognit. Emot.* **30**(5), 912–924. 10.1080/02699931.2015.1032893 (2016). 25947896 10.1080/02699931.2015.1032893

[23] Etkin, A., Egner, T., Peraza, D. M., Kandel, E. R. & Hirsch, J. Resolving emotional conflict: a role for the rostral anterior cingulate cortex in modulating activity in the amygdala. *Neuron* **51**(6), 871–882. 10.1016/j.neuron.2006.07.029 (2006). 16982430 10.1016/j.neuron.2006.07.029

[24] Lambert, H. K., King, K. M., Monahan, K. C. & McLaughlin, K. A. Differential associations of threat and deprivation with emotion regulation and cognitive control in adolescence. *Dev. Psychopathol.* **29**(3), 929–940. 10.1017/S0954579416000584 (2017). 27424571 10.1017/S0954579416000584 PMC5243929

[25] Song, S. *et al.* The influence of emotional interference on cognitive control: A meta-analysis of neuroimaging studies using the emotional Stroop task. *Sci. Rep.* **7**(1), 2088. 10.1038/s41598-017-02266-2 (2017). 28522823 10.1038/s41598-017-02266-2 PMC5437037

[26] Stroop, J. R. Studies of interference in serial verbal reactions. *J. Exp. Psychol.* **18**, 643–662 (1935). 10.1037/h0054651

[27] Pessoa, L. How do emotion and motivation direct executive control?. *Trends Cognit. Sci.* **13**(4), 160–166. 10.1016/j.tics.2009.01.006 (2009). 19285913 10.1016/j.tics.2009.01.006 PMC2773442

[28] Mitterschiffthaler, M. T. *et al.* Neural basis of the emotional Stroop interference effect in major depression. *Psychol. Med.* **38**(2), 247–256. 10.1017/S0033291707001523 (2008). 17825123 10.1017/S0033291707001523

[29] Ben-Haim, M. S. *et al.* The emotional Stroop task: assessing cognitive performance under exposure to emotional content. *JoVE (J. Visual. Exp.)* **112**, e53720. 10.3791/53720 (2016). 10.3791/53720 PMC4993290 27405091

[30] Clayson, P. E., Shuford, J., Rast, P., Baldwin, S., Weissman, D., & Larson, M. J. (2023). Normal congruency sequence effects in psychopathology: A behavioral and electrophysiological examination using a confound-minimized design. 10.1111/psyp.14426 37668221

[31] Joyal, M. *et al.* Characterizing emotional Stroop interference in posttraumatic stress disorder, major depression and anxiety disorders: A systematic review and meta-analysis. *PLoS ONE* **14**(4), e0214998. 10.1371/journal.pone.0214998 (2019). 30964902 10.1371/journal.pone.0214998 PMC6456228

[32] Krug, M. K. & Carter, C. S. Adding fear to conflict: A general purpose cognitive control network is modulated by trait anxiety. *Cognit. Affect. Behav. Neurosci.* **10**, 357–371. 10.3758/CABN.10.3.357 (2010). 20805537 10.3758/CABN.10.3.357

[33] Kray, J., Ritter, H. & Mueller, L. The interplay between cognitive control and emotional processing in children and adolescents. *J. Exp. Child Psychol.* **193**, 104795. 10.1016/j.jecp.2019.104795 (2020). 32018193 10.1016/j.jecp.2019.104795

[34] Cohen, A. O. *et al.* When is an adolescent an adult? Assessing cognitive control in emotional and nonemotional contexts. *Psychol. Sci.* **27**(4), 549–562. 10.1177/0956797615627625 (2016). 26911914 10.1177/0956797615627625

[35] Bellingtier, J. A., Luong, G., Wrzus, C., Wagner, G. G. & Riediger, M. A domain-differentiated approach to everyday emotion regulation from adolescence to older age. *Psychol. Aging* **37**(3), 338. 10.1037/pag0000677 (2022). 35084897 10.1037/pag0000677 PMC9117440

[36] Sabatier, C., Restrepo Cervantes, D., Moreno Torres, M., Hoyos De los Rios, O. & Palacio Sañudo, J. Emotion regulation in children and adolescents: Concepts, processes and influences. *Psicología desde el Caribe* **34**(1), 101–110 (2017).

[37] Faul, F., Erdfelder, E., Lang, A.-G. & Buchner, A. G*Power 3: A flexible statistical power analysis program for the social, behavioral, and biomedical sciences. *Behav. Res. Methods* **39**, 175–191 (2007). 17695343 10.3758/BF03193146

[38] Epp, A. M., Dobson, K. S., Dozois, D. J. & Frewen, P. A. A systematic meta-analysis of the Stroop task in depression. *Clin. Psychol. Rev.* **32**(4), 316–328. 10.1016/j.cpr.2012.02.005 (2012). 22459792 10.1016/j.cpr.2012.02.005

[39] Melton, T. H., Croarkin, P. E., Strawn, J. R. & Mcclintock, S. M. Comorbid anxiety and depressive symptoms in children and adolescents: a systematic review and analysis. *J. Psychiatric Pract.* **22**(2), 84. 10.1097/PRA.0000000000000132 (2016). 10.1097/PRA.0000000000000132 PMC6267783 27138077

[40] Schneider, S., Unnewehr, S. & Margraf, J. *Kinder-DIPS–Diagnostisches Interview bei Kindern und Jugendlichen mit psychischen Störungen. [Child DIPS: Diagnostic Interview for Mental Disorders in Childhood and Adolescence]* 2nd edn. (Springer, 2009). 10.13154/rub.101.90.

[41] Neuschwander, M., In-Albon, T., Adornetto, C., Roth, B. & Schneider, S. Interrater-Reliabilität des Diagnostischen Interviews bei psychischen Störungen im Kindes-und Jugendalter (Kinder-DIPS). *Z Kinder Jugendpsychiatr Psychother* **41**(5), 319–334. 10.1024/1422-4917/a000247 (2013). 23988834 10.1024/1422-4917/a000247

[42] Lundqvist, D., Flykt, A. & Öhman, A. Karolinska directed emotional faces (KDEF) [Database record]. *APA PsycTests*. 10.1037/t27732-000 (1998). 10.1037/t27732-000

[43] Helminen, E. C., Morton, M. L., Wang, Q. & Felver, J. C. A meta-analysis of cortisol reactivity to the Trier Social Stress Test in virtual environments. *Psychoneuroendocrinology* **110**, 104437. 10.1016/j.psyneuen.2019.104437 (2019). 31536942 10.1016/j.psyneuen.2019.104437

[44] Johnson, M. M. *et al.* A modified trier social stress test for vulnerable Mexican American adolescents. *JoVE (J. Visual. Exp.)* **125**, e55393. 10.3791/55393 (2017). 10.3791/55393 PMC5612053 28715387

[45] Kirschbaum, C., Pirke, K. M. & Hellhammer, D. H. The ‘Trier Social Stress Test’–a tool for investigating psychobiological stress responses in a laboratory setting. *Neuropsychobiology* **28**(1–2), 76–81. 10.1159/000119004 (1993). 8255414 10.1159/000119004

[46] Gratz, K. L. & Roemer, L. Multidimensional assessment of emotion regulation and dysregulation: Development, factor structure, and initial validation of the difficulties in emotion regulation scale. *J. Psychopathol. Behav. Assess.* **26**, 41–54. 10.1023/B:JOBA.0000007455.08539.94 (2004). 10.1023/B:JOBA.0000007455.08539.94

[47] Ehring, T., Tuschen-Caffier, B., Schnülle, J., Fischer, S. & Gross, J. J. Emotion regulation and vulnerability to depression: spontaneous versus instructed use of emotion suppression and reappraisal. *Emotion* **10**(4), 563. 10.1037/a0019010 (2010). 20677873 10.1037/a0019010

[48] Beck, A. T., Steer, R. A., & Brown, G. (1996). *Beck Depression Inventory–II* (BDI-II) [Database record]. APA PsycTests. 10.1037/t00742-000

[49] Osman, A., Kopper, B. A., Barrios, F., Gutierrez, P. M. & Bagge, C. L. Reliability and validity of the Beck depression inventory–II with adolescent psychiatric inpatients. *Psychol. Assess.* **16**(2), 120. 10.1037/1040-3590.16.2.120 (2004). 15222808 10.1037/1040-3590.16.2.120

[50] Spielberger, C. D. *Manual for the State-Trait Anxieg Inventor_y STAI (Form Y)* (Consulting Psychologists Press, 1983). 10.1037/t06496-000.

[51] Knowles, K. A. & Olatunji, B. O. Specificity of trait anxiety in anxiety and depression: Meta-analysis of the State-Trait Anxiety Inventory. *Clin. Psychol. Rev.* **82**, 101928 (2020). 33091745 10.1016/j.cpr.2020.101928 PMC7680410

[52] Sheppes, G. & Meiran, N. Divergent cognitive costs for online forms of reappraisal and distraction. *Emotion* **8**(6), 870. 10.1037/a0013711 (2008). 19102598 10.1037/a0013711

[53] Başgöze, Z., Gönül, A. S., Baskak, B. & Gökçay, D. Valence-based Word-Face Stroop task reveals differential emotional interference in patients with major depression. *Psychiatry Res.* **229**(3), 960–967. 10.1016/j.psychres.2015.05.099 (2015). 26272019 10.1016/j.psychres.2015.05.099

[54] Cheng, P. *et al.* Evidence against mood-congruent attentional bias in Major Depressive Disorder. *Psychiatry Res.* **230**(2), 496–505 (2015). 26477954 10.1016/j.psychres.2015.09.043

[55] Schweizer, S., Parker, J., Leung, J. T., Griffin, C. & Blakemore, S. J. Age-related differences in affective control and its association with mental health difficulties. *Dev. Psychopathol.* **32**(1), 329–341. 10.1017/S0954579419000099 (2019). 10.1017/S0954579419000099 PMC6982534 30907719

[56] Minihan, S. *et al.* The relationship between cognitive and affective control and adolescent mental health. *JCPP Adv.* **4**(1), e12204. 10.1002/jcv2.12204 (2024). 38486950 10.1002/jcv2.12204 PMC10933673

[57] Sætren, S. S., Augusti, E.-M. & Hafstad, G. S. Affective inhibitory control and risk for internalizing problems in adolescents exposed to child maltreatment: A population-based study. *J. Abnormal Psychol.* **130**(2), 113–125. 10.1037/abn0000582 (2021). 10.1037/abn0000582 33315413

[58] Volkaert, B., Wante, L., Wiersema, J. R. & Braet, C. Depressive symptoms in early adolescence: the dynamic interplay between emotion regulation and affective flexibility. *Front. Psychol.* **15**, 1165995. 10.3389/fpsyg.2024.1165995 (2024). 38586295 10.3389/fpsyg.2024.1165995 PMC10996852

[59] Straub, J. *et al.* Adolescent depression and brain development: Evidence from voxel-based morphometry. *J. Psychiatry Neurosci.* **44**(4), 237–245. 10.1503/jpn.170233 (2019). 30720261 10.1503/jpn.170233 PMC6606428

[60] Jasielska, A. *et al.* The relationship between working memory and emotion regulation strategies. *Roczniki Psychologiczne* **18**(4), 567–578. 10.18290/rpsych.2015.18.4-4en (2019). 10.18290/rpsych.2015.18.4-4en

[61] O’Bryan, E. M., Kraemer, K. M., Johnson, A. L., McLeish, A. C. & McLaughlin, L. E. Examining the role of attentional control in terms of specific emotion regulation difficulties. *Personal. Individ. Differ.* **108**, 158–163. 10.1016/j.paid.2016.12.015 (2017). 10.1016/j.paid.2016.12.015

[62] Gyurkovics, M., Stafford, T. & Levita, L. Cognitive control across adolescence: Dynamic adjustments and mind-wandering. *J. Exp. Psychol. Gen.* **149**(6), 1017. 10.1037/xge0000698 (2020). 31599622 10.1037/xge0000698

[63] Luna, B., Padmanabhan, A. & O’Hearn, K. What has fMRI told us about the development of cognitive control through adolescence?. *Brain Cognit.* **72**(1), 101–113. 10.1016/j.bandc.2009.08.005 (2010). 19765880 10.1016/j.bandc.2009.08.005 PMC2815087

[64] Solmi, M. *et al.* Age at onset of mental disorders worldwide: Large-scale meta-analysis of 192 epidemiological studies. *Mol. Psychiatry* **27**(1), 281–295. 10.1038/s41380-021-01161-7 (2022). 34079068 10.1038/s41380-021-01161-7 PMC8960395

[65] Holmes, A. J. & Pizzagalli, D. A. Response conflict and frontocingulate dysfunction in unmedicated participants with major depression. *Neuropsychologia* **46**(12), 2904–2913. 10.1016/j.neuropsychologia.2008.05.028 (2008). 18577391 10.1016/j.neuropsychologia.2008.05.028 PMC2538441

[66] Shimony, O. *et al.* The association between implicit and explicit affective inhibitory control, rumination and depressive symptoms. *Sci. Rep.* **11**(1), 11490. 10.1038/s41598-021-90875-3 (2021). 34075112 10.1038/s41598-021-90875-3 PMC8169859

[67] Paulus, M. P. Cognitive control in depression and anxiety: out of control?. *Curr. Opin. Behav. Sci.* **1**, 113–120. 10.1016/j.cobeha.2014.12.003 (2015). 10.1016/j.cobeha.2014.12.003

[68] Chen, S. & Bonanno, G. A. Components of emotion regulation flexibility: linking latent profiles to depressive and anxious symptoms. *Clin. Psychol. Sci.* **9**(2), 236–251. 10.1177/2167702620956972 (2021). 10.1177/2167702620956972

[69] Wang, X., Blain, S. D., Meng, J., Liu, Y. & Qiu, J. Variability in emotion regulation strategy use is negatively associated with depressive symptoms. *Cogn. Emot.* **35**(2), 324–340. 10.1080/02699931.2020.1840337 (2021). 33150844 10.1080/02699931.2020.1840337

[70] Godara, M., Everaert, J., Sanchez-Lopez, A., Joormann, J. & De Raedt, R. Interplay between uncertainty intolerance, emotion regulation, cognitive flexibility, and psychopathology during the COVID-19 pandemic: a multi-wave study. *Sci. Rep.* **13**(1), 9854. 10.1038/s41598-023-36211-3 (2023). 37330557 10.1038/s41598-023-36211-3 PMC10276821

[71] McKone, K. M., Edershile, E. A., Ladouceur, C. D. & Silk, J. S. Real-world flexibility in adolescent girls’ emotion regulation strategy selection: An investigation of strategy switching. *Dev. Psychopathol.* **36**(1), 181–195. 10.1017/S0954579422001079 (2024). 36503633 10.1017/S0954579422001079 PMC10258216

[72] Bardeen, J. R. & Fergus, T. A. Emotion regulation self-efficacy mediates the relation between happiness emotion goals and depressive symptoms: A cross-lagged panel design. *Emotion* **20**(5), 910. 10.1037/emo0000592 (2020). 30816743 10.1037/emo0000592

[73] Orth, U., Robins, R. W. & Roberts, B. W. Low self-esteem prospectively predicts depression in adolescence and young adulthood. *J. Personal. Soc. Psychol.* **95**(3), 695. 10.1037/0022-3514.95.3.695 (2008). 10.1037/0022-3514.95.3.695 18729703

[74] Blanke, E. S., Bellingtier, J. A., Riediger, M. & Brose, A. When and how to regulate: Everyday emotion-regulation strategy use and stressor intensity. *Affect. Sci.* **3**(1), 81–92. 10.1007/s42761-021-00087-1 (2022). 36042783 10.1007/s42761-021-00087-1 PMC9382984

[75] Dixon-Gordon, K. L., Aldao, A. & De Los Reyes, A. Emotion regulation in context: Examining the spontaneous use of strategies across emotional intensity and type of emotion. *Personal. Individ. Differ.* **86**, 271–276. 10.1016/j.paid.2015.06.011 (2015). 10.1016/j.paid.2015.06.011

[76] Lande, N. M., Ask, T. F., Sætren, S. S., Lugo, R. G. & Sütterlin, S. The role of emotion regulation for general self-efficacy in adolescents assessed through both neurophysiological and self-reported measures. *Psychol. Res. Behav. Manag.* **16**, 3373–3383. 10.2147/PRBM.S406 (2023). 37650113 10.2147/PRBM.S406 PMC10464900

[77] Vasilev, C. A., Crowell, S. E., Beauchaine, T. P., Mead, H. K. & Gatzke-Kopp, L. M. Correspondence between physiological and self-report measures of emotion dysregulation: A longitudinal investigation of youth with and without psychopathology. *J. Child Psychol. Psychiatry* **50**(11), 1357–1364. 10.1111/j.1469-7610.2009.02172.x (2009). 19811585 10.1111/j.1469-7610.2009.02172.x

