# Supplementary material for: The role of affective control, strategy repertoire and subjective emotion regulation success in developmental internalising psychopathology
Source: Sci Rep. 2024 Sep 11;14:21224. doi: 10.1038/s41598-024-72336-9 (PMC11390718; doi:10.1038/s41598-024-72336-9)
Supplement: Supplementary file 1 — Supplementary Information. [file 41598_2024_72336_MOESM1_ESM.docx]

Supplementary materials:

**The role of affective control, strategy repertoire and subjective emotion regulation success in developmental internalising psychopathology**

Carolin C. L. M. Herber¹*, Lea L. Lott-Sandkamp¹, Elisa R. Straub² & Brunna Tuschen-Caffier¹*

*¹ Department for Clinical Psychology and Psychotherapy, University of Freiburg, Germany*

*² Department for General Psychology, University of Freiburg, Germany*

**Supplementary methods**

**Deviations from the preregistration**

Three changes were made in this paper that deviate from what was done in the preregistration. Firstly, the DV affective control was operationalised in the preregistration as the “incongruency effect”. What is meant by this term is the congruency effect but the terms were confused due to the effect relying upon the difference of the congruent from the incongruent trials. We later on realised that only looking at affective control as measured by the congruency effect is quite limiting because it only entails the difference between these two scores, it does not actually say anything about the participants RT or error rate. This is why in the process of analysing the data and talking to researchers that are more familiar with the EST, we decided to look at affective control more broadly and highlight its multiple components by also taking the mean RT and error rate as measures of affective control. Secondly, we preregistered to do an ANOVA for H1 because this is how the EST is often analysed (Cohen & Henik, 2012). We later on realised though that since we had defined affective control to be made up of three facets, it would be best to instead do a MANOVA. This choice then also led us to do another power calculation of a MANOVA instead of an ANOVA, which constitutes the third change that was made from the preregistration.

**Table S1**

*Specification of given disorders and their frequency per clinical group*

|  | |  |  |  |
| --- | --- | --- | --- | --- |
| Group Affiliation | Disorder |  | N (Primary disorder) | N (Secondary disorder) |
| Depression | Major depressive disorder |  | 13 | 0  0 |
|  | Persistent depressive disorder |  | 4 |  |
| Anxiety | Social anxiety disorder |  | 7 | 10 |
|  | Panic disorder  Generalised anxiety disorder  Specific phobia  Other (Insomnia, OCD) |  | 3  5  1  0 | 9  4  4  5 |

*Note.* Insomnia = Insomnia Disorder, OCD = Obsessive Compulsive Disorder

**Figure S1**

*Flow chart of the laboratory session*


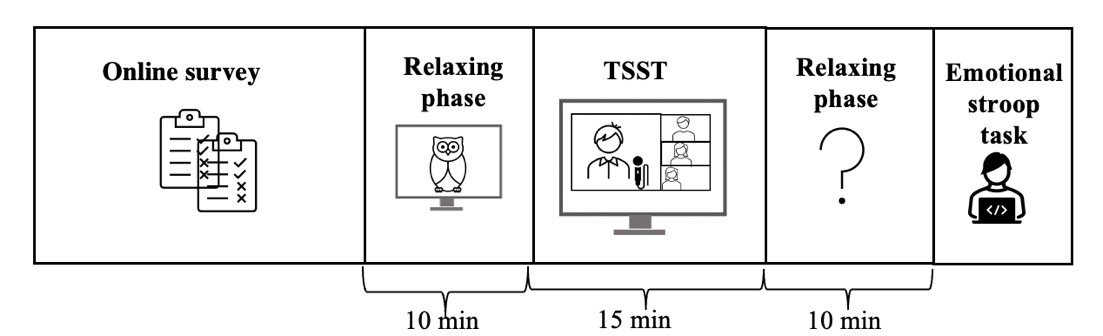


*Note.* The overall length of session 2 was approximately 1.5 hours and varied depending on how long participants took to fill out the online survey.

**Table S2**

*Self-report list of emotion regulation (ER) strategies used*

| **ER strategy** | **Description** |
| --- | --- |
| Cognitive reappraisal | “I reminded myself that the Zoom Call was only part of this study and that I do not have to care about how I did, since I will never see the jurors again.” |
| Acceptance | “I tried to accept the situation as it is: Accept that I am stressed right now, because I know that I will feel better over time.” |
| Distraction | “I tried to distract myself to not have to think about the Zoom Call.” |
| Social Support | “I talked or texted with someone who I know would make me feel better.” |
| Problem solving | “I tried to get more information on the Zoom Call, its purpose and use from the experimenter to get mental clarity.” |
| Mindfulness | “I tried to focus on my breathing and the sensations in my body in order to calm down.” |
| Other | “I did something else that is not descripted above in order to regulate my stress.” |

**Figure S2**

*Flowchart of procedures: Screening process, allocation and analysis*

**
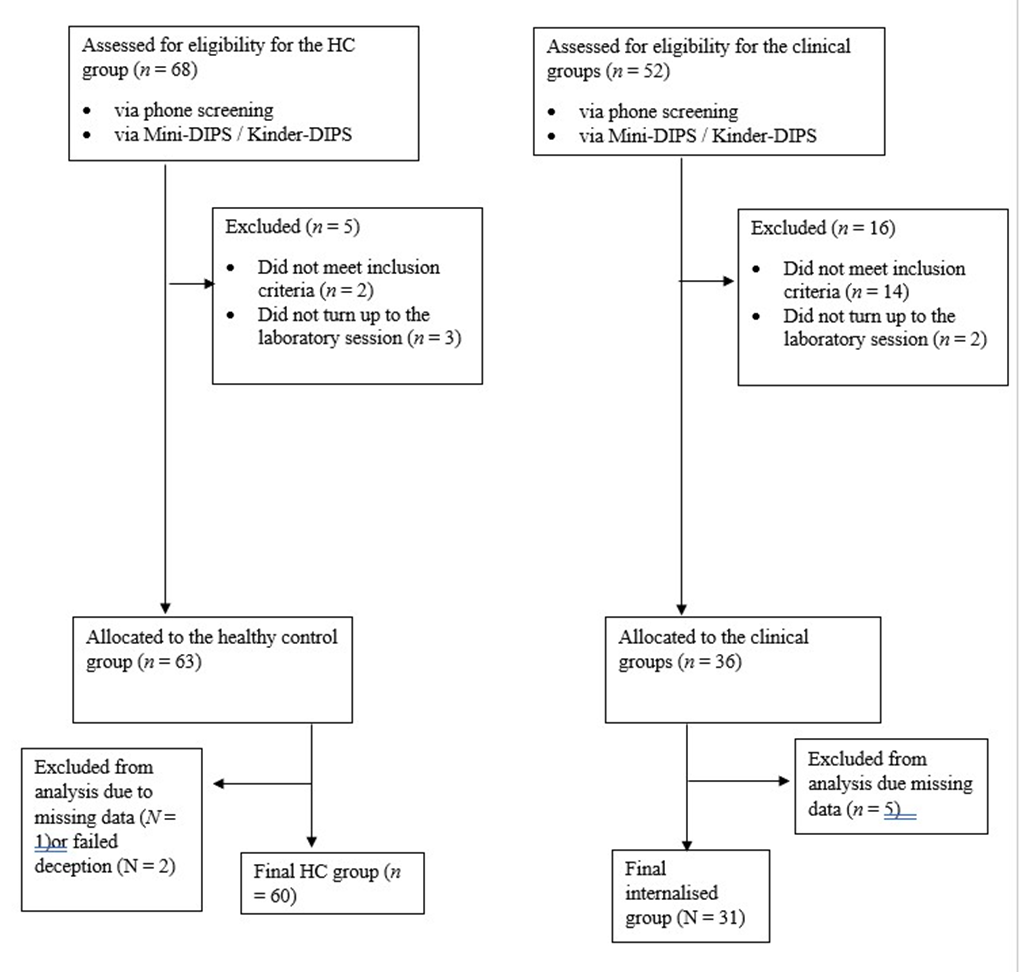
**

*Note*. Due to fact that more participants from the clinical group were excluded due to not meeting inclusion criteria (e.g. comorbidities, suicidality, use of psychoactive medication), the clinical group size ended up smaller than initially planned (N = 60) and could not be reached by the end of the recruitment period.

**Figure S3**

*Flow chart of the TSST for adolescents*

*
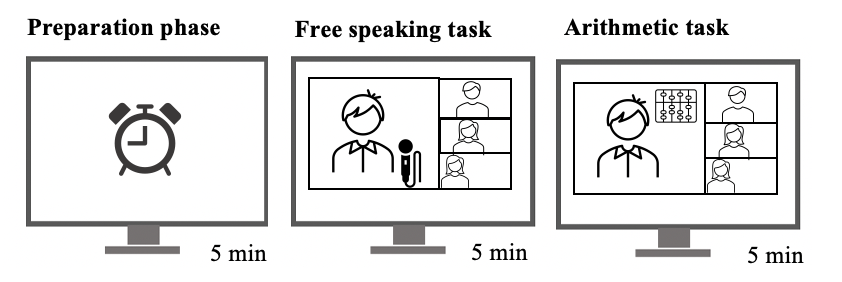
*

*Note.* The TSST instructions were based on the protocol of Johnson et al., (2017).

**Manipulation check of the stress induction using the digital TSST**

A one-tailed, within-subjects t-test revealed a significant increase in heart rate measured for eight minutes per time frame between the baseline level (*M* = 75.8, *SD* = 10.98) and the level after the stress test (*M* = 93.88, *SD* = 16.44), *t*(80) = 62.12, *p* < .001. There was also a significant increase in the subjective stress level between the baseline level (*M* = 2.53, *SD* = 1.4) and the level after the stress test (*M* = 4.87, *SD* = 1.78), *t*(91) = 17.32, *p* < .001.

**Supplementary results**

**Table S3**

Descriptive Statistics of the error rate and the congruency effect between healthy young adults, healthy adolescents, young adults with internalised disorder and adolescents with internalised disorders

|  | **Group** | **Age group** | ***M*** | ***SD*** | ***N*** |
| --- | --- | --- | --- | --- | --- |
| Error rate | HC | Young adults | 8.64 | 5.17 | 33 |
|  |  | Adolescents | 17.3 | 11.37 | 18 |
|  | CLIN | Young Adults | 10.66 | 7.69 | 13 |
|  |  | Adolescents | 16.94 | 9.8 | 19 |
|  | Total | Young Adults | 9.21 | 5.97 | 46 |
|  |  | Adolescents | 17.12 | 10.45 | 37 |
| Congruency effect | HC | Young Adults | 44.81 | 48 | 33 |
|  |  | Adolescents | 46.35 | 42.88 | 18 |
|  | CLIN | Young Adults | 30.97 | 46.06 | 13 |
|  |  | Adolescents | 39.29 | 40.27 | 19 |
|  | Total | Young Adults | 40.9 | 47.37 | 46 |
|  |  | Adolescents | 42.72 | 41.13 | 37 |

**Table S4**

*Descriptive statistics of RTs in the EST (M* ± *SD*)

|  | CLIN group (n = 31) | HC group (n = 60) |
| --- | --- | --- |
| All correct trials | 565.24 ± 96.1 | 571.79 ± 84.43 |
| Congruent trials |  |  |
| Happy faces | 539.13 ± 91.51 | 539.54 ± 92.51 |
| Fearful faces | 561.89 ± 104.3 | 558.07 ± 85.18 |
| Total | 547.28 ± 96.94 | 549.11 ± 85.66 |
| Incongruent trials |  |  |
| Happy faces | 583.42 ± 106.63 | 582.45 ± 90.17 |
| Fearful faces | 593.89 ± 102.87 | 598.89 ± 99.12 |
| Total | 583.19 ± 89.27 | 594.46 ± 89.28 |

**Manipulation check of the EST**

Descriptive statistics of RTs in the EST per group, trial type (congruent vs. incongruent) and emotion (happy vs. fearful) can be found in the supplementary materials. To test whether the EST did in fact produce a congruency effect, we computed a mixed model ANOVA (Greenhouse-Geisser correction) with congruency (congruent vs. incongruent) and emotion (happy vs. fearful) as within-subject factors, group as a between-subjects factor and sex as a covariate. The analysis revealed significant main effects of congruency, *F* (1, 88) = 5.42, *p* = .022, $\eta_{p}^{2}$=.06, and emotion, *F* (1, 88) = 9.24, *p* = .003, $\eta_{p}^{2}$=.12, while there was no significant main effect of group. Bonferroni-adjusted pairwise comparisons revealed significantly (*p* < .001) shorter mean RT of congruent trials than incongruent trials (*M*_Diff_ = 16.16, 95%-CI[7.57, 24.74]) and significantly (*p* < .001) shorter mean RT of happy than fearful faces (*M*_Diff_ = 38.49, 95%-CI[28.92, 48.06]). Furthermore, the two-way interaction congruency×emotion was significant, *F* (1, 88) = 6.84, *p* = .010, $\eta_{p}^{2}$=.07. A post-hoc pairwise comparison revealed significantly (p < .001) longer RTs for fearful than happy faces for both congruent trials (*M*_Diff_ = 42.23, 95%-CI[28.84, 55.63]) and incongruent trials (*M*_Diff_ = 43.75, 95%-CI[22.42, 47.08]). In addition, the three-way interaction congruency×emotion×sex was significant, *F* (1, 88) = 5.8, *p* = .018, $\eta_{p}^{2}$=.06.

**Figure S4**

*EST performance across groups by congruency and emotion*

*Note.* Since there was no significant main effect of group, the figure shows bars for congruency and emotion but not by group. Error bars depict the standard deviation of the mean.

**Moderator analysis of H4.** Moderation analysis was conducted using SPSS’s PROCESS macro (Hayes, 2013). The interaction between trait ER difficulties and age was not significant (*R*^2^= .24, *F*(3, 87) = 1.88, *p* = .139), indicating that the relationship between trait ER difficulties and affective control was not moderated by age.

**Legend**

**Table S1.** Specification of given disorders and their frequency per clinical group

**Figure S1.** Flow chart of the laboratory session

**Table S2.** Self-report list of emotion regulation (ER) strategies used

**Figure S2.** Flowchart of procedures: Screening process, allocation and analysis

**Table S3.** Descriptive Statistics of the error rate and the congruency effect between healthy young adults, healthy adolescents, young adults with internalised disorder and adolescents with internalised disorders

**Table S4.** Descriptive statistics of RTs in the EST (M ± SD)

**Figure S4.** EST performance across groups by congruency and emotion
